# Editorial: Plant-based diets for a sustainable future

**DOI:** 10.3389/fnut.2023.1342174

**Published:** 2024-01-17

**Authors:** Cecília Medeiros de Morais, Rui Poínhos, Aslı Uçar

**Affiliations:** ^1^Department of Biology and the Environment, School of Life and Environmental Sciences, Universidade de Trás-os-Montes e Alto Douro, Vila Real, Portugal; ^2^GreenUPorto, Sustainable Agrifood Production Research Centre, Porto, Portugal; ^3^Faculdade de Ciências da Nutrição e Alimentação da Universidade do Porto [Faculty of Nutrition and Food Sciences, University of Porto], Porto, Portugal; ^4^Department of Nutrition and Dietetics, Faculty of Health Sciences, Ankara University, Ankara, Türkiye

**Keywords:** plant-based diet, sustainability, health, nutrition, food

In an era when our choices profoundly impact the planet, the way we eat takes on new significance. Plant-based diets have emerged as a sustainable solution for both our health and the environment. This issue delves into the world of plant-based diets, exploring their health benefits and their role in creating a sustainable future.

The human diet continually evolves, initially relying solely on vegetable foods collected from nature and transitioning to plant-based diets with the introduction of agriculture. Throughout civilizations, preferences for plant-based foods are shaped by religion, culture, health, personal choices, and economic factors (1). Plant-based diets are associated to vegetarianism or other diets that naturally promote and priorize the consumption of foods from vegetable sources and can be more or less flexible in the inclusion of animal foods. These include flexitarian, semi-vegetarian, pesco-vegetarian, lacto-ovo-vegetarian and vegan diets (2). Additionally, they may align with traditional and intricate dietary styles like the Mediterranean Diet, Nordic Diet, or DASH (2). Some of the reasons for not eating animal-based foods may be related to personal, cultural and religious beliefs mostly focused on animal wellbeing, but can also emanate from the loss of confidence on animal-based products, which have affected the food choices of the modern consumers (1). Following the tendency for plant-based diets, the meat substitute products are a promising option and an alternative source for protein intake. Still, Garaus and Garaus found that US consumers' predominantly negative perceptions of meat substitutes, identifying gender-based differences and consumer profiles, offering insights to help market and promote plant-based alternatives more effectively.

In practice, we can analyse the nutritional challenges and the possible health gains of plant-based diets. First, comparing plant-based with meat-based diets, the nutritional intake of the first was found to be lower for vitamin B12, vitamin D and iodine, but also for calcium, eicosapentaenoic acid (EPA) and docosahexaenoic acid (DHA). The average intake of iron and zinc were also considered inadequate since their requirements are higher due to the lower bioavailability presented from the vegetable sources. Nevertheless, the meat-based was higher for fiber, polyunsaturated fatty acids (PUFA), α-linolenic acid (ALA), vitamin E, folate, and magnesium (3). But if this is true for adults, in contrast to children, adolescents, pregnant or lactating women, and older adults, authors tend to be more cautious (2). Another focus for concern is the adequacy of dietary protein and aminoacid intake from plant-based diets. Recent studies in adults following classic vegetarian diets indicate they can supply protein and aminoacids above the body requirements (4). A recent study investigating the ideal percentage of protein in plant diets and its correlation with nutrition and sustainability found no singular optimal value, proposing a broad range of 25–70%. The study revealed that a well-balanced plant-based diet, rich in high-quality proteins and diverse plant foods, could potentially safeguard against bone loss. Conversely, an unhealthy plant-based diet might adversely affect bone mineral density (BMD). Despite potential limitations such as reliance on self-reported dietary data, the research suggests that meticulously planned vegetarian diets merit further exploration for their potential bone health benefits across diverse populations (Fouillet et al.).

Precisely, we can consider that the nutritional adequacy may be compromised for the people following plant-based diets but also for the general population mainly if they follow unhealthy food habits, with a lack of food variety but rich in processed foods. It can also be critical specifically for the more vulnerable population groups and the ones with specific and higher requirements, such as the older adults or athletes, and the populations from poor countries where the minimum requirements are compromised (1–3). Nutrition education strategies, along with food fortification may be an important means to fight against nutrition deficiencies and malnutrition.

Nutritional adequacy can also be affected by the food composition, which is variable in terms of the different nutrients depending on many factors from soil and agriculture to the intrinsic genotypic variations of plants, or food innovation and processing technologies. The study highlights that certain Indian onion cultivars, especially the yellow bulb “Arka Pitamber,” have a high potassium-to-sodium ratio, which could be beneficial in dietary interventions to manage hypertension and reduce cardiovascular disease risks in the Indian population (Singh et al.). Modern techniques for food production can work as an ally on the creation of foods with more interesting nutrient profiles for certain conditions or pathologies. Shi et al. ‘s study aims to transform spent barley grains into a nutritious ingredient for making starch less noodles with minimal impact on blood sugar, presenting a sustainable food option particularly suitable for individuals with diabetes and those concerned about blood sugar and weight management.

Following the advances of Epidemiology but also the achievements of clinical and basic science, since the beginning of the twenty-first century a shift has occurred and the benefits for health from a plant-based diet has become stronger based on evidence, with particular attention to the management of chronic diseases risk, the lower mortality rates and the increased longevity (1–3, 5). Noteworthy, we still don't have consistent conclusions as the health benefits associated to plant-based diets are still a matter for debate (2, 5). Although some studies did not find any association or benefits on vegetarian diet for weight management (3), studies focusing on physiological changes found that vegan diets reduced body weight (6) and a clinical trial found these diets were more effective for weight loss (7). More recently, Magkos et al. concluded there was still limited evidence that the severe restriction of meat-based foods will impact on the reduction of overweight and obesity (2). Ghadiri et al. found that among Iranian postmenopausal women with osteopenia/osteoporosis, adherence to a healthy plant-based diet is associated with a lower likelihood of bone mineral density abnormalities, whereas an unhealthy plant-based diet correlates with a significantly increased risk of such abnormalities. Analyzing the benefits from more balanced diets, such as DASH, Mediterranean and Nordic diets, the associations to longevity and health are well-known, namely in terms of lower mortality and lower risk for non-communicable diseases (2). Several studies also indicated that plant-based diets were associated with lower risk for cancer, for type 2 diabetes and cardiovascular diseases (2, 8), However, a stronger evidence to support these arguments is still necessary (2). More frequently, people choose plant-based or popular diets for health reasons, and usually those individuals are more interested in nutritional rationale, but the study from Jontez et al.'s study compares the nutrient intake and serum metabolic biomarkers of individuals on self-selected diets like LCHF, vegan, vegetarian, and omnivorous, finding that while LCHF adherents consume comparable micronutrients, their higher intake of saturated fats and cholesterol, and lower intake of fibers, may necessitate healthier fat sources and more plant-based foods to optimize nutrient levels.

More recently, plant-based diets have been associated with a need to save the planet and promote the environmental causes. A sustainable diet should ensure a good nutritional status and health of individuals. It should also have a low environmental impact and contribute to the health and wellbeing of future generations. This concept comprises a range of very different dimensions, including nutritional, social, ecological, and economical (2).

Mannucci et al. examines the complexity of sustainable nutrition with a focus on vegetable oils, emphasizing the need for interdisciplinary research and holistic approaches to address the health, environmental, and socio-economic factors in food production and consumption for future global food security.

The planet is exceeding its limits and “Planetary health” is at risk, with serious adverse effects, such as climate change, decrease in freshwater reserves, the loss of biodiversity and changes at the biogeochemical flows (8). The unprecedented growth of the world population over the past 70 years, soaring from 2.5 to 8 billion, has intensified the challenge of ensuring a sustainable and nutritious food supply for the global population. One of the reasons for having more sustainable diets is that they can contribute to the reduction of the greenhouse gases (GHG) emissions. But if the traditional food production and food patterns have an impact at the planetary health, the opposite is also true, with recent problems related to the impact of natural disasters or adverse climatic conditions at food production. In general, the main findings reveal that plan-based diets are more sustainable. It is also important to analyse the impact of the different foods and not only the type of diet since there is a high variability regarding environmental impact for each food, even if they are part of the same diet (8). Another issue to discuss is the cost of foods that have a smaller environmental footprint, since the higher prices are a limitation for the populations with lower economic resources (1). Rochefort et al. found that among French Canadian adults, diets with lower animal-based and higher plant-based protein intakes are associated with better diet quality and are more cost-effective, suggesting the benefits of such diets for health and sustainability. Following a sustainable and healthy diet was also associated to a better knowledge about sustainable nutrition and environmental responsible choices (Yassibaş and Bölükbaşi), which points the challenge of improving the populations literacy concerning this Research Topics. Nevertheless, Macit-Çelebi et al. revealing a low compliance with EAT-Lancet recommendations that is associated with higher obesity rates, highlighting the need for education to improve diet sustainability. The studies mentioned above collectively reinforce the idea that our dietary choices are not just about personal health but also about the health of our planet. As we move toward a more sustainable future, plant-based diets may hold the key to achieving both personal and environmental wellness. It's worth mentioning that more research and continued awareness are necessary to fully embrace plant-based diets and make them an integral part of our sustainable future. As individuals, we can take steps toward a more plant-centric diet, and as a society, we can support policies and practices that promote the sustainability of our food choices. By doing so, we contribute to a healthier and more sustainable future for ourselves and generations to come.

## Author contributions

CM: Writing—original draft. RP: Writing—review & editing. AU: Writing—review & editing.
